# Screening of pregnant women for foetal neonatal alloimmune thrombocytopenia: A cost–utility analysis

**DOI:** 10.1111/vox.13779

**Published:** 2024-12-05

**Authors:** Thijs W. de Vos, Ilonka Tersteeg, Enrico Lopriore, Dick Oepkes, Leendert Porcelijn, C. Ellen van der Schoot, E. Joanne T. Verweij, Dian Winkelhorst, Masja de Haas, M. Elske van den Akker‐van Marle

**Affiliations:** ^1^ Willem‐Alexander Children's Hospital, Department of Pediatrics, Division of Neonatology Leiden University Medical Center Leiden the Netherlands; ^2^ Department of Experimental Immunohematology Sanquin Research Amsterdam the Netherlands; ^3^ Department of Biomedical Data Sciences, Section Medical Decision Making Leiden University Medical Center Leiden the Netherlands; ^4^ Department of Obstetrics and Gynecology Leiden University Medical Center Leiden the Netherlands; ^5^ Department of Immunohematology Diagnostics Sanquin Diagnostic Services Amsterdam the Netherlands; ^6^ Department of Hematology Leiden University Medical Center Leiden the Netherlands

**Keywords:** cost–effectiveness analysis, foetal and neonatal alloimmune thrombocytopenia, HPA‐1a screening

## Abstract

**Background and Objectives:**

Foetal and neonatal alloimmune thrombocytopenia (FNAIT) results from maternal platelet‐directed antibodies and can result in severe intracranial haemorrhage (ICH) in foetuses and newborns. Screening for human platelet antigen‐1a (HPA‐1a)‐directed antibodies during pregnancy could allow timely intervention with antenatal treatment and prevent ICH. We assessed the cost effectiveness of HPA‐1a typing and anti‐HPA‐1a‐screening as part of the prenatal screening programme.

**Materials and Methods:**

Different HPA‐1a screening scenarios were tested in a decision analysis model and assessed for diagnostic, treatment, intervention and lifetime costs and prevention effects compared to the current situation without screening in the Netherlands. Model parameters were based on available data, literature and expert opinions. One‐way sensitivity analysis and probabilistic sensitivity analysis were performed.

**Results:**

Adding screening for anti‐HPA‐1a antibodies to the current antenatal screening programme of the Netherlands will lead to an additional cost of €4.7 million per year and a gain of 226 quality‐adjusted life years (QALYs) per year, indicating an incremental cost–effectiveness ratio (ICER) of €20,782 per QALY gained. One‐way sensitivity analysis showed that the uncertainty around the incidence of ICH, lifetime costs of disabled children and the probability of having antibody quantitation >3.0 IU/mL at 20 weeks had the highest effect on the ICER.

**Conclusion:**

Antenatal anti‐HPA‐1a screening might be cost effective. To obtain more knowledge and thereby to improve risk stratification, a pilot screening programme is warranted.

**Highlights**

- Antenatal anti‐human platelet antigen‐1a (HPA‐1a) screening might be cost effective within the European healthcare system.
- Screening for anti‐HPA‐1a antibodies in pregnancy demands consensus on risk stratification, for example, based on HPA‐1a antibody quantification.
- Knowledge about the effectiveness of antenatal treatment with intravenous immunoglobulins in first high‐risk foetal and neonatal alloimmune thrombocytopenia pregnancies is limited, and the effectiveness of any HPA‐1a‐antibody screening programme should therefore be further investigated in a randomized trial.

## INTRODUCTION

Foetal and neonatal alloimmune thrombocytopenia (FNAIT) is a rare but severe disease that may cause intracranial haemorrhage (ICH) and organ bleeding in foetuses and neonates. FNAIT results from maternal IgG antibodies directed against paternally inherited antigens on the foetal platelets. In the population of European descent, the majority of FNAIT cases are caused by antibodies directed against human platelet antigen 1a (HPA‐1a) [1]. Implementation of population‐based screening for FNAIT, in analogy to red blood cell antibody screening for secondary prevention of severe haemolytic disease of the foetus and neonate, has been debated for decades [2, 3, 4]. It is argued that by screening, HPA‐1a alloimmunized pregnancies can be identified and that timely antenatal intervention could prevent the occurrence of ICH and its life‐long neurological sequelae [2, 3, 4, 5, 6, 7, 8]. In line with previous studies, we found in our recent study on the natural history of FNAIT that 3722 of 153,106 (2.4%) of the pregnant women were HPA‐1a negative, with 85 of 913 (9.3%) of these women HPA‐1a antibody positive as tested in Week 27 of pregnancy [9].

Over the last decades, several cost–effectiveness (CE) studies on HPA screening have been performed [5, 6, 7, 8]. In a study published in 1988, Gafni and Blanchette [5] performed a hypothetical calculation assuming that anti‐HPA‐1a prophylaxis would prevent all FNAIT‐related morbidity; however, such prophylaxis is not yet available, although the first pre‐clinical and clinical studies have shown promising results [10, 11]. A study published in 1996 by Durand‐Zaleski et al. [6] focused only on postnatal screening, making optimal management of a next pregnancy possible, whereas later it became apparent that foetal ICH cases are also already diagnosed during first ongoing pregnancies [12]. In 1998, Williamson et al. [13] proposed to select high‐risk pregnancies with serial antibody measurements during pregnancy because primigravida women may produce clinically relevant HPA‐1a antibodies during pregnancy and in multigravida women antibody levels may decline to non‐relevant quantities [13]. Based on these insights, Turner et al. [7] calculated the diagnostics test costs for antenatal screening; however, their study had a relatively limited sample size. Finally, Killie et al. [8] performed a CE study based on a large screening study [14] with the assumption that near‐term caesarean section and the immediate availability of HPA‐1a‐negative donor platelets for the severely thrombocytopenic babies would prevent the development of ICH.

In concordance with both Williamson et al. [13] and Killie et al. [15], we propose for a Dutch screening programme to identify HPA‐1a‐negative and HLA‐DRB3*0101‐positive women and to screen for HPA‐1a antibodies at Weeks 20 and 27. To select high‐risk pregnancies and start treatment with intravenous immunoglobulins (IVIg), we propose to use an HPA‐1a antibody level. We assessed the cost versus effectiveness of various scenario's of an anti‐HPA‐1a screening programme compared to the current situation without screening.

## MATERIALS AND METHODS

We developed a decision analysis model in Microsoft Excel and compared the lifetime costs and effects of antenatal anti‐HPA‐1a screening with the situation without screening, using costs of tests and treatments in the Netherlands. Because the proposed screening programme will impact both the life expectancy and quality of life of children with FNAIT, the outcome was expressed in quality‐adjusted life years (QALYs). The incremental cost–effectiveness ratio (ICER) was expressed in terms of incremental cost per QALY. We assessed the costs and consequences of screening from a societal perspective: that is, all costs and consequences were included regardless of who incurs the costs and who obtains the effects. Costs have been discounted at a constant rate of 4% and effects at a constant rate of 1.5% [16]. The price level of 2022 was used. Calculations were based on a population of 171,713 pregnant women [17]. Since the consequences of ICH can result in lifelong handicaps [18], we applied a lifetime horizon.

### Probabilities

#### Situation without antenatal HPA‐1a screening

**FIGURE 1:**
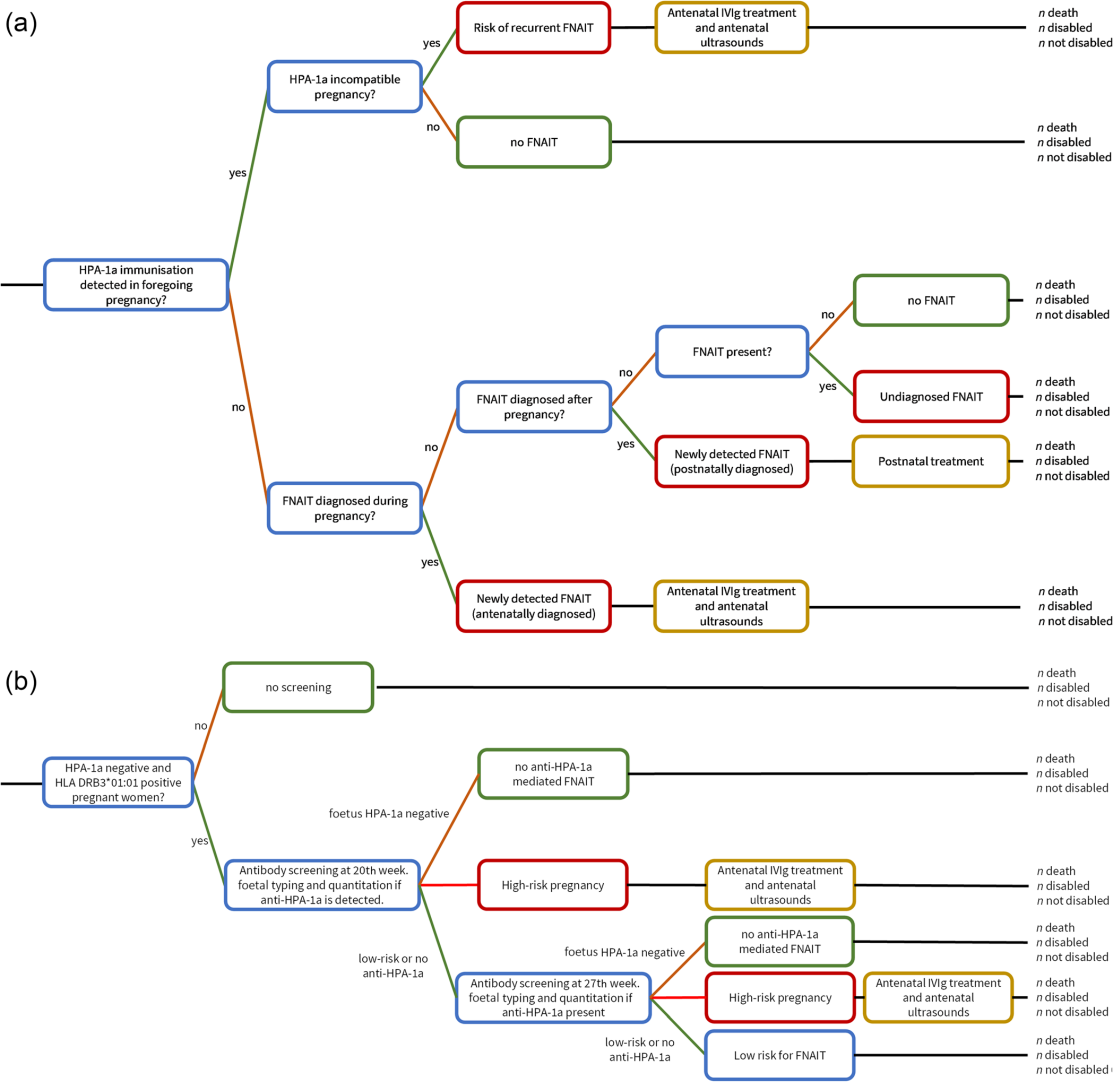
Diagram of the situation without human platelet antigen‐1a (HPA‐1a) screening and the situation with HPA‐1a screening. (a) without screening. (b) with screening. FNAIT, foetal and neonatal alloimmune thrombocytopenia; HLA, human leukocyte antigen; IVIg, intravenous immunoglobulins.

Figure 1a (decision tree in Figure S1) shows the situation without HPA‐1a‐antibody screening. In the absence of screening, HPA‐1a negativity is rarely known in pregnancy and FNAIT is highly underdiagnosed [19]. In the base case, the probability of HPA‐1a‐associated ICH due to undiagnosed FNAIT (5.5 cases/year in the Netherlands) was based on data from a recent screening study in the Netherlands [9] and a systematic review [20].

In the situation without screening, the probability of giving birth to a child diagnosed with HPA‐1a‐mediated FNAIT postnatally (9.3 cases/year in the Netherlands, of which 0.9 cases/year diagnosed with ICH [21]) was based on a previous study in the Netherlands (2002–2019) [21]. Probabilities on the postnatal outcome (e.g., platelet count) of newly diagnosed FNAIT cases were retrieved from an international multicentre study [22].

A minority of antenatally IVIg‐treated FNAIT cases is diagnosed during pregnancy upon detection of ICH by ultrasound (1 case/year in the Netherlands [21]). Outcome of children with ICH was estimated on a case series of 21 children with FNAIT‐related ICH: 52% died, 33% were alive and had neurodevelopmental impairment (classified as disabled) and 14% were alive without neurodevelopmental impairment (classified as not disabled) [18].

Lastly, based on data collected from antenatally IVIg‐treated follow‐up pregnancies after a previous child diagnosed with FNAIT, we assumed no disability in this group of children (estimated on 4.2 cases/year in the Netherlands) [21, 23]. All probabilities of the situation without screening are listed in Table S1.

#### Antenatal screening

The situation with anti‐HPA‐1a screening is shown in Figure 1b (decision tree in Figure S2). All pregnant women will be typed for HPA‐1a early in pregnancy and subsequently for HLA‐DRB3*01:01, because women negative for HLA DRB3*01:01 rarely develop high levels of anti‐HPA‐1a [24]. HPA‐1a‐antibody screening will be offered at the 20th and 27th gestational week. Foetal HPA‐1a typing will be performed to confirm foetal–maternal incompatibility. High‐risk or low‐risk pregnancies will be discriminated by HPA‐1a‐antibody quantitation according to the cut‐off values based on the Norwegian screening study [15]. If antibody quantitation is >3 IU/mL, the pregnancy is considered high risk, and the mother is treated by weekly administration of IVIg (dosage; 0.5 g/kg/week).

The proportion of HPA‐1a negativity (2.4%) is based on the results of a recent Dutch screening study [9]. The probability of being HLA DRB3*01:01‐positive (33%) is based on data of two cohorts of healthy blood donors [25, 26]. Data on the course of antibody quantitation and probabilities of having antibody quantitation >3 IU/mL at 20th week and/or 27th week were based on the Norwegian screening study [15]. Probabilities of the situation with HPA screening are shown in Table S2.

### Costs

#### Diagnostics test costs

Costs of diagnostic tests are shown in Table S3. In the no‐screening situation, FNAIT is diagnosed with maternal, paternal and neonatal (molecular) comprehensive HPA‐1, ‐2, ‐3, ‐5 and ‐15 typing, HPA and HLA antibody identification and cross‐matching paternal platelets with maternal serum (€1953). Costs of testing in the situation without screening were based on the current prices of the National Reference Laboratory [27, 28]. In case of an anti‐HPA‐1a screening programme, test costs for the then more routinely applied testing will be lowered and will be comparable for the programme run for red blood cell antibody detection. Costs for diagnostic tests in a screening setting were calculated by diagnostic experts on platelet antibody screening from Sanquin (M.d.H. and L.P.). Costs used for the screening setting in the base case were €15 for HPA‐1 phenotyping (including costs for logistics and the reports), €40 for HLA‐DRB3*01:01 typing by array technology, €75 for HPA‐antibody screening and €150 for anti‐HPA‐1a quantitation.

#### Treatment costs

Treatment costs are presented in Table S3. Antenatal treatment costs consist of both administration costs and medication costs for weekly administration of IVIg to the pregnant woman (€223 per vial of 2.5 g [29]). Every first IVIg dosage during pregnancy is given in the hospital on day‐care basis (€304 [30]), and subsequent dosages are administered by home‐care nurses (€200 per administration [personal communication former Sanquin, currently Prothya, home service]). Additionally, costs for healthcare resource use were calculated, including outpatient clinic visits [30] with costs of advanced foetal ultrasounds (€851 [31]) at 21, 27, 31 and 35 weeks gestational age. These costs were calculated as additional costs compared to healthcare costs in the situation without screening. Travel costs and productivity costs of pregnant women were also taken into account.

Types of cost of postnatal treatment to the newborn with FNAIT depend on neonatal platelet counts, which were categorized into three groups. Neonates with platelet count >100 × 10^9^/L are regarded not at risk for bleeding and discharged; no additional costs were calculated for this group. Neonates with a platelet count 25–100 × 10^9^/L will be admitted for clinical surveillance to the maternity ward (3 days, €449 per day [32]) including daily measurements of platelet counts. In addition, cranial ultrasound (€100 [32]) will be performed to screen for ICH. Neonates with platelet count <25 × 10^9^/L will be admitted to the neonatology ward (high care, €1830 per day [32]) and receive one HPA‐matched platelet transfusion (€365). In addition, brain imaging and platelet count measurements will be done. Healthcare‐related and travel costs that might be attributable to the father were not included in this analysis. No loss of productivity costs were applied because postnatal treatment falls within the period of maternity leave.

#### Lifetime costs per health state

Additional lifetime costs related to FNAIT per health state are shown in Table S5. Additional lifetime costs for the outcomes healthy, not disabled or death were set at €0. Literature on the lifetime costs for FNAIT‐related disability is lacking, and therefore we used reports on lifetime costs of cerebral palsy (CP). We used data from a study from Denmark [33] that reported on the lifetime costs for healthcare, productivity costs and societal costs for children with CP (€802,868 excluding informal costs). Productivity costs were subtracted from these lifetime costs, as in this study the friction cost approach is used [30]. According to this approach, disabled children do not account for productivity costs because they never entered and therefore will never leave the labour market. Costs for informal caregiving (€341,000) were based on a study reporting on the mean hours of informal care per week for severe neurological conditions [34] and the cost per hour of caregiving [30].

### Effects

In Tables S5 and S6, utility values, reflecting the quality of life within a particular health state, are shown. No data were available on health‐related quality of life related to FNAIT. One study systematically assessed the long‐term outcome of children with FNAIT‐related ICH and reported that 70% had CP, 40% had severe visual impairment and 40% were diagnosed with epilepsy [18]. Therefore, literature on the utility scores of children diagnosed with CP [35], visual impairment [36, 37] and epilepsy [38] was used. Based on the available literature, the utility score of FNAIT‐related disability was estimated at 0.55. A utility score of 0 was assigned to ‘death’ as health state. For the healthy and not disabled health state, the Dutch population norm score was used (0.910) [39]. Life expectancy of disabled children was assumed to be 50 years [40] and for children not disabled 81.7 years [41].

### Assumptions

In the base case, we assumed no failure of antenatal treatment. We also assumed that all cases at risk for FNAIT‐related ICH develop antibodies with levels >3 IU/mL at the 27th week or earlier.

### Analyses

#### Base‐case analysis

A base‐case analysis was performed by using the values for the model parameters described above. We reported costs, QALYs, FNAIT‐related death and FNAIT‐related disability for the situation without screening and the situation with antenatal HPA‐1a screening. To calculate the ICER, difference in mean costs between the situation with and without antenatal screening is divided by the difference in mean QALYs.

A cost–utility ratio was calculated, because it allows comparison across different health programmes and policies by using a common unit of measure. Furthermore, the resulting cost–utility ratio can be compared with the societal willingness‐to‐pay threshold for a country, to judge whether an intervention is cost effective compared to usual care.

#### Sensitivity analyses

One‐way sensitivity analysis (OWSA) and probabilistic sensitivity analysis (PSA) were performed to address the uncertainty of the model parameters and to quantify the impact on costs and QALYs. To perform these analyses, beta, gamma and Dirichlet distributions were used around the parameters. Beta distribution was applied to all parameter values that needed to stay within the 0–1 range, thus for the probabilities and utilities. Gamma distribution applies to parameters that are not allowed to drop below 0 (e.g., costs, or the annual number of pregnant women). A Dirichlet distribution was chosen when a parameter consisted of more than two proportional parameters that had to add up to 1 every time. Ranges of these distributions were based on expert opinion (T.d.V. and M.d.H.). For the beta and gamma distributions, either a standard error has been assumed or values for alpha and beta were estimated in line with the assumed minimum and maximum value of the parameter. Assumptions about the standard error (SE) were made in collaboration with the experts, taking a percentage of the deterministic value depending on how much variation was considered likely. For the costs related to the disabled health state, for example, an SE of 50% was assumed because these costs are expected to show much variation.

OWSA included all probabilities except the parameters with Dirichlet distribution. The 15 parameters with the largest effect on the ICER were presented in a Tornado diagram. PSA was performed by random draws from the probability distribution for 1000 simulations. Subsequently, costs and QALYs were calculated for each simulation. Results for this analysis were displayed in a CE plane and cost effectiveness acceptability curve (CEAC).

#### Scenario analysis 1: Newborn platelet counts as quality control

In the first years after the introduction of HPA‐1a screening, quality control will be performed to verify if clinically relevant FNAIT cases will be left untreated. In this scenario analysis, platelet counts will be performed in all neonates of HPA‐1a‐negative women to assess extra costs of this quality control and benefits introduced by this measurement.

#### Scenario analysis 2: Higher antibody threshold as risk stratification

In the base‐case analysis, women are considered to have a high‐risk pregnancy if antibody quantitation is >3 IU/mL. However, severe cases usually have higher HPA‐1a antibody levels [9, 13, 14, 15, 42, 43, 44], and we therefore performed a scenario analysis in which we set the threshold at 10 IU/mL.

#### Scenario analysis 3: Reduced sensitivity of screening

Antibody quantitation is doubted as single predictor for disease severity because in retrospective studies, cases were identified with ICH and low antibody levels [43]. To address this uncertainty, we performed a scenario analysis in which yearly 1 out of 194 pregnancies classified as low risk at 27 weeks gestational age ended with the delivery of a child with ICH.

#### Scenario analysis 4: Reduced effectivity of IVIg treatment

In a fourth scenario, it was presumed that in 20% of pregnancies IVIg is not effective and the risk of ICH would be equal to the risk in the group with unidentified FNAIT.

### Ethics statement

This study did not involve human or animal subjects, or medical records; therefore, ethical approval was not applicable.

## RESULTS

### Base‐case analysis

Results of the base‐case analysis with an annual number of 171,713 pregnant women are shown in Table 1. Incorporating these annual expected numbers and the effect assumptions, an expected yearly number of 2.5 children with FNAIT‐related disability and 3.8 cases of FNAIT‐related death were obtained for the Netherlands in a situation without screening.

**TABLE 1:** Disaggregated results and increments compared to no screening situation for a cohort of 171,713 pregnant women (2022).

Category	No screening	HPA‐1a screening	Incremental screening versus no screening
Annual number of deaths caused by FNAIT	3.83	0.00	–3.83
Annual number of disabilities caused by FNAIT	2.48	0.00	–2.48
Total QALYs attained (discounted)	7,208,369	7,208,595	+226
Diagnostic test costs	€26,200	€3,042,100	+€3,015,900
Antenatal treatment costs	€252,400	€4,630,200	+€4,377,800
Postnatal treatment costs	€66,800	€201,600	+€134,800
Lifetime costs	€2,840,400	€0	–€2,840,400
Total costs	€3,185,800	€7,873,900	+€4,688,100

Abbreviations: FNAIT, foetal and neonatal alloimmune thrombocytopenia; QALYs, quality‐adjusted life years.

In the situation with antenatal screening, we expect to identify 64.7 high‐risk pregnancies at 20th week of pregnancy and 10.7 high‐risk pregnancies at 27th week of pregnancy per year. Because of the earlier HPA‐antibody detection and antenatal treatment, we expect to prevent all FNAIT‐related disability and death: a yearly gain of 226 QALYs is expected (discounted). The number of women needed to be treated antenatally with IVIg (number needed to treat, NNT) is 10.2 (75.4 of 7.4) to prevent one ICH. Total annual cost increase of HPA‐1a screening expected was €4,688,100. Dividing the difference in costs by the 226 QALYs gained resulted in a cost–utility ratio of €20,782 per QALY gained.

### Sensitivity analysis

Results of the OWSA are presented in Figure S3. In this analysis, we changed the base‐case parameters to their minimum and maximum values (Tables S1–S4). The uncertainty around the exact incidence of HPA‐1a‐mediated ICH in the group with currently unidentified FNAIT, lifetime costs of disabled children and the probability of having antibody quantitation >3.0 IU/mL at 20 weeks of gestation had the highest impact on the ICER.

**FIGURE 2:**
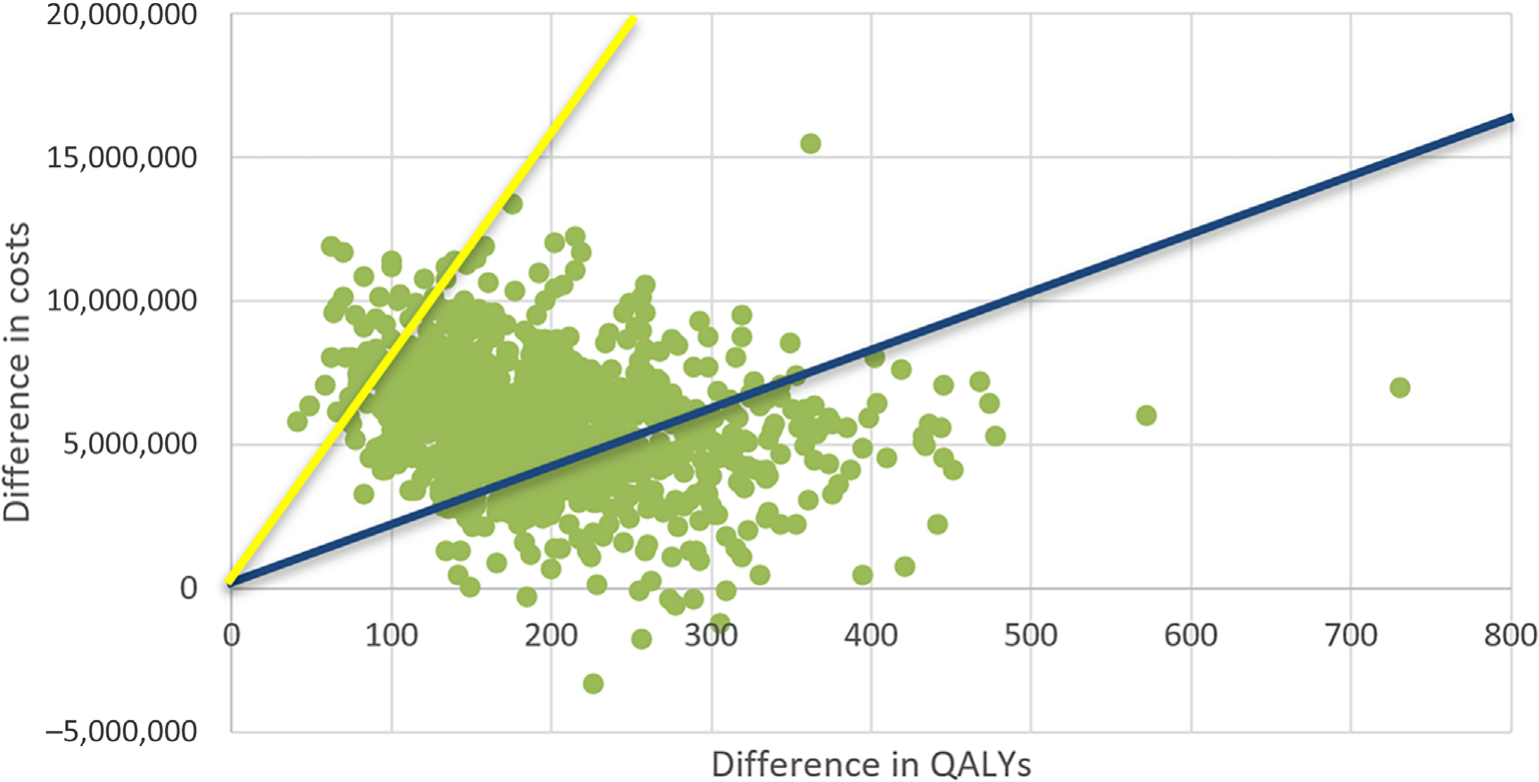
Probabilistic sensitivity analysis: Cost–effectiveness plane. Cost effectiveness is based on 1000 probabilistic simulations. The blue line represents the €20,000 per quality‐adjusted life year (QALY) threshold, and the yellow line represents the €80,000 per QALY threshold.

**FIGURE 3:**
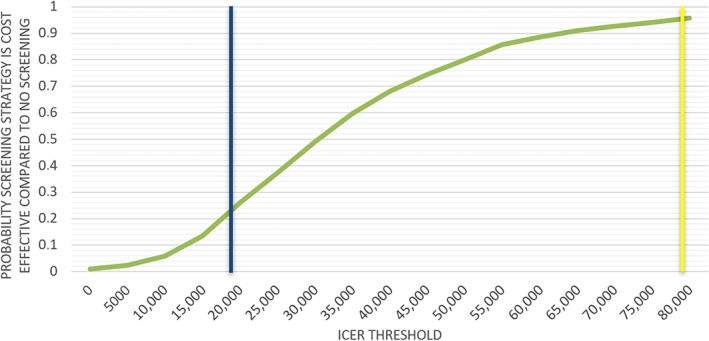
Probabilistic sensitivity analysis: Cost–effectiveness acceptability curve. Cost effectiveness is based on 1000 probabilistic simulations. The blue line represents the €20,000 per quality‐adjusted life year (QALY) threshold, and the yellow line represents the €80,000 per QALY threshold. ICER, incremental cost–effectiveness ratio.

In addition, a PSA was performed (Figures 2 and 3). At a willingness‐to‐pay threshold of €20,000 per QALY, the probability of the screening strategy being cost effective compared to a situation without screening was 26%. At a willingness‐to‐pay threshold of €80,000, this percentage was 96%.

### Scenario analyses

The base‐case analysis was chosen conservatively; with the design, we aimed to include pregnancies at risk of FNAIT, and a quality control of the screening programme to evaluate if HPA‐1a‐mediated neonatal thrombocytopenia was missed. Four different scenarios were used to evaluate how a change in design of the base‐case analysis would impact costs and utility of the screening programme.

In Scenario 1, we observed that performing platelet counts in all HPA‐1a‐positive newborns from HPA‐1a‐negative mothers as a quality control of the screening programme would lead to an yearly additional cost of €26,387. It was observed that it would not lead to improved performance of a screening programme, because it did not prevent costs or improved QALY. An isolated thrombocytopenia—without bleeding tendency—in newborns does not cause immediate or long‐term health impairment. It may lead to detection of alloimmunization in the mother, which may have occurred shortly before delivery, but this will also be noted in a subsequent pregnancy. Therefore, the costs to check platelet counts in all newborns of HPA‐1a‐negative women can be prevented.

In Scenario 2, we used a higher threshold of HPA‐1a quantity for risk stratification. Selection of high‐risk pregnancies with antibody quantitation of >10 IU/mL would lead to considerable reduction in costs. The cost increment will be €2,930,164 instead of €4,688,103. It is, however, currently uncertain to what extent this might lead to missing cases at risk for ICH. Every missed ICH would lead to lower QALYs and higher costs.

Therefore, in Scenario 3, we assessed the impact of one missing case yearly with ICH. This would lead to a reduction in the gain of QALYs to only 192 instead of 226. And a cost–utility ratio of €26,559 per QALY will be gained. This is 28% higher compared to the base‐case analysis.

Finally, 100% efficacy of antenatal treatment with IVIg was assumed in the base‐case analysis. If the efficacy of IVIg treatment would be lower (Scenario Analysis 4), a gain of only 183 QALY was expected, resulting in a cost–utility ratio of €28,483 per QALY gained. This is a rise of 37%.

## DISCUSSION

In the base‐case analysis, we found that the addition of HPA‐1a‐antibody screening to the current antenatal screening programme of the Netherlands will lead to an additional cost of €4.7 million and a gain of 226 QALY per year. Thus, the ICER was €20,782 per QALY gained. OWSA showed that the uncertainty around the incidence of ICH in the group of unidentified FNAIT, lifetime costs of disabled children and the probability of having antibody quantitation >3.0 IU/mL at 20 weeks of gestation had the highest effect on the ICER.

Turner et al. [7] calculated $71,067 (€84,747 price level 2022) per QALY gained. This higher amount can be possibly explained by the fact that this study focused exclusively on costs for diagnostic testing without taking the costs and savings for treatment and prevention of disability into account. Therefore, the effect in reduction of lifetime treatment costs was not included, resulting in a higher CE ratio. Killie et al. [8] calculated that all screening strategies, including a near‐term caesarean section as preventive measure [14], were cost‐saving. Whether this approach indeed would reduce FNAIT‐related severe antenatal bleeding has, however, been questioned [45]. Killie et al. calculated that HPA‐1a typing and antibody screening of a population of 100,000 pregnant women would result in 210–230 gained QALYs (discounted rate) [8]. This was higher than in our study (132 QALYs per 100,000 HPA‐1a‐ and HLA‐DRB3*01:01‐typed and subsequently antibody‐screened pregnant women).

In line with the conclusions of Killie et al., the CE ratio found in our study is also acceptable for European countries [46]. In addition, further cost reductions are possible. At present, as part of the national prevention programme in pregnancy to prevent haemolytic disease of the foetus and newborn, maternal blood group typing (ABO, RhD, Rhc) is repeated in every pregnancy. In our current design of a screening programme to prevent FNAIT, we followed the same approach with repeated testing in each pregnancy. However, all antenatal testing, including red blood cell antigen typing, HPA‐1a and HLA typing, could easily be made accessible in a central database and used for subsequent pregnancies, thus avoiding unnecessary re‐testing and costs.

A driver in the programme for prevention of haemolytic disease of the foetus and newborn is the administration of anti‐D to RhD‐negative women. Although anti‐HPA‐1a prophylaxis is currently being developed [10], data on its effectiveness are not available. It is hoped that it will be as successful as anti‐D prophylaxis. In a scenario with anti‐HPA‐1a prophylaxis, costs for detection of HPA‐1a and HLA typing and anti‐HPA‐1a antibody screening remain; the number of alloimmunized women may decline, and thus less costs for treatment can be expected [47]. We did not consider new treatment options with FcRn‐inhibiting monoclonal antibody therapy that may become available [48].

Our study has several limitations. Most importantly, our study was based on the assumption that IVIg treatment could prevent all anti‐HPA‐1a‐related ICH and that all immunizations leading to ICH will be detected in a screening strategy. It is, however, unknown if IVIg also reduces the risk of bleeding in first HPA‐1a‐immunized pregnancies. It is also not known if all types of HPA‐1a antibodies are pathogenic to similar extents [49, 50]. Therefore, it may be advised to start a screening programme in a study set‐up with a control group to obtain more knowledge on both the effectiveness of IVIg treatment and risk‐stratification options, possibly also testing additional HPA‐1a antibody characteristics next to antibody level quantitation [49, 50]. Before such a trial can be conducted, extensive ethical consideration should be given to the study design. On one hand, it is clear that IVIg treatment is not necessary in every HPA‐1a‐immunized pregnancy [51]. Administration of a rare, donor‐derived, immune‐modulating and costly treatment should be applied only if necessary. On the other hand, antenatal IVIg treatment has shown effectiveness in second HPA‐1a‐immunized pregnancies; hence, how to prevent unjustified withholding of the therapy? Finally, it may be that development of a high bleeding tendency is multifactorial, and additional genetic risk factors increase the risk of bleeding, also at relatively low anti‐HPA‐1a titres [52].

Another limitation is that healthcare costs in the Netherlands may not account for costs in other settings. Acknowledging the limitations of our study, we do agree with previous studies that HPA‐1a screening in pregnancy has the potential to be cost effective. It is therefore of utmost importance to allow risk stratification within the group of HPA‐1a‐alloimmunized pregnant women and to restrict IVIg therapy to women with a high risk of having a child with ICH, and it should be taken into account that it may not be possible to prevent ICH in every HPA‐1a‐alloimmunized pregnancy.

## CONFLICT OF INTEREST STATEMENT

D.O. is funded as a research consultant by Janssen Pharmaceuticals Inc. and participates on the Advisory Board of Janssen Pharmaceuticals Inc. E.L. reports a consultancy fee from Janssen Pharmaceuticals Inc. as member of the Advisory Board on FNAIT. The other authors report no conflicts of interest.

## Supporting information


**Data S1.** Supporting information.

## Data Availability

The model used to conduct the cost–effectiveness analysis is available via the corresponding author after all co‐authors agree to share the model.
