# Supplementary material for: Screening of pregnant women for foetal neonatal alloimmune thrombocytopenia: A cost–utility analysis
Source: Vox Sang. 2024 Dec 5;120(2):178–87. doi: 10.1111/vox.13779 (PMC11839253; doi:10.1111/vox.13779)
Supplement: Supplementary file 1 — Data S1. Supporting information. [file VOX-120-178-s001.docx]

# Supplemental Tables

## Supplemental Table 1: Probabilities used in situation “without HPA-1a screening”.

| **Parameter** | **Probability** | **Distribution**  **Beta (SE) or**  **Dirichlet (n1, n2, n3,..)** | **Source** |
| --- | --- | --- | --- |
| *General* | | | |
| Termination of pregnancy / fetal loss during pregnancy | 0.033 | Beta (0.002)† | Process monitor of the Dutch prenatal screening programme for infectious disease and erythrocyte immunisation^13^ |
| *Probabilities of pregnancies of women who were diagnosed with HPA-1a immunization in previous pregnancy* | | | |
| Pregnant woman diagnosed with FNAIT in foregoing pregnancy | 2.459 × 10^-5^ | Beta (4.918 × 10^-6^) § | Nationwide FNAIT database^18^ |
| Fetus HPA-1a positive if FNAIT was diagnosed in foregoing pregnancy | 0.844 | Beta (0.042) † | Calculated based on data from a Dutch prospective screening study (HIP study) ^4, 16^ |
| False-negativity rate fetal HPA-1a typing | 0.030 | Beta (0.003) ‡ | Assumed equal to fetal *RHD* typing. ^48^ |
| Fetal loss due to failure of antenatal treatment | 0.000 | Dirichlet (1,1700,1400,1000) | Expert opinion |
| PC > 100 × 10^9^/L after antenatal treatment | 0.415 | Dirichlet (1,1700,1400,1000) | FNAIT registry 2020^19^ |
| PC 25-100 × 10^9^/L after antenatal treatment | 0.341 | Dirichlet (1,1700,1400,1000) |
| PC < 25 × 10^9^/L after antenatal treatment | 0.244 | Dirichlet (1,1700,1400,1000) |
| Dead if PC > 100 × 10^9^/L | 0.000 | Dirichlet (1,10,999989) | Expert opinion |
| Disabled if PC > 100 × 10^9^/L | 0.000 | Dirichlet (1,10,999989) |
| Not disabled if PC > 100 × 10^9^/L | 1.000 | Dirichlet (1,10,999989) |
| Dead if PC 25-100 × 10^9^/L | 0.000 | Dirichlet (1,10,99989) |
| Disabled if PC 25-100 × 10^9^/L | 0.000 | Dirichlet (1,10,99989) |
| Not disabled if 25-100 × 10^9^/L | 1.000 | Dirichlet (1,10,99989) |
| Dead if PC < 25 × 10^9^/L | 0.000 | Dirichlet (1,5,94) |
| Disabled if PC < 25 × 10^9^/L | 0.000 | Dirichlet (1,5,94) |
| Not disabled if PC < 25 × 10^9^/L | 1.000 | Dirichlet (1,5,94) |
| *Probabilities if FNAIT is diagnosed in current pregnancy* | | | |
| FNAIT detected during current pregnancy | 6.022 × 10^-6^ | Beta (9.218 × 10^-6^) | Nationwide FNAIT database ^18^ |
| Termination of pregnancy/IUFD due to FNAIT | 0.800 | Beta (0.160)|| |
| Fetal loss due to failure of antenatal treatment | 0.000 | Dirichlet (2,1,1,100) | Expert opinion |
| PC > 100 × 10^9^/L after antenatal treatment | 0.000 | Dirichlet (2,1,1,100) |
| PC 25-100 × 10^9^/L after antenatal treatment | 0.000 | Dirichlet (2,1,1,100) |
| PC < 25 × 10^9^/L after antenatal treatment | 1.000 | Dirichlet (2,1,1,100) |
| Dead if PC < 25 × 10^9^/L after antenatal treatment | 0.000 | Dirichlet (1,10,90) |
| Disabled if PC < 25 × 10^9^/L after antenatal treatment | 0.100 | Dirichlet (1,10,90) |
| Not disabled if PC < 25 × 10^9^/L after antenatal treatment | 0.900 | Dirichlet (1,10,90) |
| *Probabilities if FNAIT is diagnosed postnatally* | | | |
| FNAIT detected after birth | 5.601 × 10^-5^ | Beta (5.601 × 10^-6^) ‡ | Nationwide FNAIT database ^18^ |
| PC > 100 × 10^9^/L | 0.000 | Dirichlet (1,300,940) |
| PC 25-100 × 10^9^/L | 0.242 | Dirichlet (1,300,940) |
| PC < 25 × 10^9^/L | 0.758 | Dirichlet (1,300,940) |
| Dead if PC 25-100 × 10^9^/L | 0.000 | Dirichlet (1,10,99989) | Expert opinion |
| Disabled if PC 25-100 × 10^9^/L | 0.000 | Dirichlet (1,10,99989) |
| Not disabled if 25-100 × 10^9^/L | 1.000 | Dirichlet (1,10,99989) |
| Death if PC < 25 × 10^9^/L after postnatal diagnosis | 0.021 | Dirichlet (20,84,836) | 14, 18 |
| Disabled if PC < 25 × 10^9^/L after postnatal diagnosis | 0.089 | Dirichlet (20,84,836) |
| Not disabled if PC < 25 × 10^9^/L after postnatal diagnosis | 0.889 | Dirichlet (20,84,836) |
| *Probabilities of unidentified FNAIT* | | | |
| Unidentified FNAIT | 3.613 × 10^-4^ | Beta (7.227 × 10^-5^)|| | 4, 17 |
| ICH due to unidentified FNAIT | 0.092 | Beta (0.018)|| |
| Dead due to ICH | 0.524 | Dirichlet (11,7,3) | 9, 14 |
| Disabled due to ICH | 0.333 | Dirichlet (11,7,3) |
| Not disabled despite ICH | 0.143 | Dirichlet (11,7,3) |
| † SE of 5%. ‡ SE of 10%. § SE of 15%. || SE of 20%. # SE of 50%.  HPA, human platelet antigen; FNAIT, fetal and neonatal alloimmune thrombocytopenia; HIP study; HPA screening in pregnancy study; PC, Platelet count; IUFD, intrauterine fetal demise; ICH, intracranial haemorrhage. | | | |

## Supplemental Table 2: Probabilities used in situation “with HPA-1a screening”

| **Parameter** | **Probability** | **Distribution**  **Beta (SE) or**  **Dirichlet (n1, n2, n3,..)** | **Reference** |
| --- | --- | --- | --- |
| *General* | | | |
| Termination of pregnancy / fetal loss during pregnancy | 0.033 | Beta (0.002)† | Process monitor of the Dutch prenatal screening programme for infectious disease and erythrocyte immunisation^13^ |
| *Maternal typing first trimester* | | | |
| HPA-1a negative pregnant women | 0.024 | Beta (0.002)‡ | Dutch prospective screening study (HIP study) ^4, 16^ |
| Women HLA DRB3*01:01 positive | 0.330 | Beta (0.017)† | Cohort from DISIII ^24^ and BloodTyper study. ^23^ |
| Maternal HPA-1a typing false negative | 0.035 | Beta (0.003)‡ | 49 |
| *Antibody screening at 20 weeks’ GA* | | | |
| Anti-HPA-1a detected | 0.232 | Beta (0.023)‡ | Dutch prospective screening study (HIP study) ^4, 16^ |
| Fetus HPA-1a positive if mother is HPA-1a immunised (and DBR3*01:01 positive) | 0.896 | Beta (0.045)† | Dutch prospective screening study (HIP study) ^4, 16^ |
| False-negative fetal HPA-1a typing | 0.030 | Beta (0.003)‡ | Assumed equal to fetal *RHD* typing. ^48^ |
| Antibody quantitation > 3 IU/ml at 20 weeks GA. (High risk pregnancy) | 0.242 | Beta (0.048) # | Dutch prospective screening study (HIP study) ^4, 16^ and ^22^ |
| *Antibody screening at 27 weeks’ GA* | | | |
| Antibodies present at 27 weeks GA but < 3.0 IU/ml at 20 weeks GA. | 1.000 | Beta (N/A)  alpha=40, beta=1 | 22 |
| Pregnancy at high risk for FNAIT when antibodies are detected at 27 weeks GA when considered low-risk at 20 weeks GA | 0.040 | Beta (0.008) # | Dutch prospective screening study (HIP study) ^4, 16^ and ^22^ |
| Dead after being considered at low risk for FNAIT (no antenatal treatment) | 0.000 | Dirichlet (1,10,99989) | Expert opinion and ^22^ |
| Disabled after being considered at low risk for FNAIT (no antenatal treatment) | 0.000 | Dirichlet (1,10,99989) |
| Not disabled after being considered at low risk for FNAIT (no antenatal treatment | 1.000 | Dirichlet (1,10,99989) |
| PC > 100 × 10^9^/L after being considered at low risk for FNAIT (no antenatal treatment) | 1.000 | Dirichlet (9989,10,1) |
| PC 25-100 × 10^9^/L after being considered at low risk for FNAIT (no antenatal treatment) | 0.000 | Dirichlet (9989,10,1) |
| PC < 25 × 10^9^/L after being considered at low risk for FNAIT (no antenatal treatment) | 0.000 | Dirichlet (9989,10,1) |
| Dead after no antibodies were detected (no antenatal treatment) | 0.000 | Dirichlet (1,10,999989) |
| Disabled after no antibodies were detected (no antenatal treatment) | 0.000 | Dirichlet (1,10,999989) |
| Not disabled after no antibodies were detected (no antenatal treatment) | 1.000 | Dirichlet (1,10,999989) |
| PC > 100 × 10^9^/L if no antibodies were detected (no antenatal treatment) | 1.000 | Dirichlet (99989,10,1) |
| PC 25-100 × 10^9^/L if no antibodies were detected (no antenatal treatment) | 0.000 | Dirichlet (99989,10,1) |
| PC < 25 × 10^9^/L if no antibodies were detected (no antenatal treatment) | 0.000 | Dirichlet (99989,10,1) |
| Fetus HPA-1a positive in HPA-1a negative mother in case antibodies are detected at 27 weeks’ GA if were absent at 20 weeks’ GA | 1.000 | N/A |
| Antibodies present at 27 weeks’ GA if were absent at 20 weeks’ GA | 0.020 | Beta (0.002) ‡ | Dutch prospective screening study (HIP study) ^4, 16^ and ^22^ |
| Pregnancy at high risk for FNAIT when antibodies are detected at 27 weeks’ GA if absent at 20 weeks’ GA | 0.132 | Beta (0.026) # |
| *Outcome after antenatal treatment* | | | |
| Fetal loss due to failure of antenatal treatment | 0.000 | Dirichlet (1,1700,1400,1000) | Expert opinion |
| PC > 100 × 10^9^/L after antenatal treatment | 0.415 | Dirichlet (1,1700,1400,1000) | FNAIT registry 2020^19^ |
| PC 25-100 × 10^9^/L after antenatal treatment | 0.341 | Dirichlet (1,1700,1400,1000) |
| PC < 25 × 10^9^/L after antenatal treatment | 0.244 | Dirichlet (1,1700,1400,1000) |
| Dead if PC > 100 × 10^9^/L | 0.000 | Dirichlet (1,10,999989) | Expert opinion |
| Disabled if PC > 100 × 10^9^/L | 0.000 | Dirichlet (1,10,999989) |
| Not disabled if PC > 100 × 10^9^/L | 1.000 | Dirichlet (1,10,999989) |
| Dead if PC 25-100 × 10^9^/L | 0.000 | Dirichlet (1,10,99989) |
| Disabled if PC 25-100 × 10^9^/L | 0.000 | Dirichlet (1,10,99989) |
| Not disabled if 25-100 × 10^9^/L | 1.000 | Dirichlet (1,10,99989) |
| Dead if PC < 25 × 10^9^/L | 0.000 | Dirichlet (1,5,94) |
| Disabled if PC < 25 × 10^9^/L | 0.000 | Dirichlet (1,5,94) |
| Not disabled if PC < 25 × 10^9^/L | 1.000 | Dirichlet (1,5,94) |
| † SE of 5%. ‡ SE of 10%. § SE of 15%. || SE of 20%. # SE of 50%.  HPA, human platelet antigen; SE, standard error; HIP study, HPA screening in pregnancy study; HLA, human leukocyte antigen; DIS, Donor InSight; FNAIT, fetal and neonatal alloimmune thrombocytopenia; IU, international units; ml, milliliter; GA, gestational age; PC, Platelet count; L, litre. | | | |

## Supplemental Table 3: Diagnostic test costs.

| **Parameter name** | **Value** | **Distribution**  **Gamma (SE)** | **Source** |
| --- | --- | --- | --- |
| *Situation without HPA-1a screening* | | | |
| Fetal HPA-1 typing in alloimmunised women | €1345.23 | NA | Sanquin Diagnostic Services ^25^ |
| Lab work-up to detect FNAIT in fetus or neonate | €1953.66 | NA | Sanquin Diagnostic Services ^26^ |
| Platelet count | €22.39 | Gamma (2.24) ‡ | 50 |
| Order rate | €9.01 | NA | 50 |
| *With HPA-1a screening* | | | |
| Maternal HPA-1 typing | €15.00 | Gamma (0.75) † | Sanquin Diagnostics Services (calculated by LP and MdH) |
| Fetal HPA-1 typing in screening setting | €43.00 | Gamma (2.15) † |
| HLA DRB3*01:01 test | €40.00 | Gamma (8.00) || |
| HPA-1a antibody screening | €75.00 | Gamma (3.75) † |
| Risk assessment (antibody quantitation) | €150.00 | Gamma (7.50) † |
| Platelet count | €22.39^∞^ | Gamma (2.24) ‡ | 50 |
| Order rate | €9.01^∞^ | NA | 50 |
| † SE of 5%. ‡ SE of 10%. § SE of 15%. || SE of 20%. # SE of 50%.  SE, standard error; HPA, human platelet antigen; NA not applicable; FNAIT, fetal and neonatal alloimmune thrombocytopenia; HLA, human leukocyte antigen. | | | |

## Supplemental Table 4: Costs.

| **Parameter name** | **Value** | **Distribution**  **Gamma (SE)** | **Source** |
| --- | --- | --- | --- |
| *Antenatal treatment* | | | |
| NaCl 500 ml 0.9% | €2.13 | Gamma (0.11) † | Medicijnkosten.nl^27^ |
| IVIg 0.1g/ml, 25 ml vial | €223.45 | Gamma (11.17) † | Medicijnkosten.nl^27^ |
| IVIg administration in hospital | €304.46 per administration | Gamma (60.89) || | Manual for cost research ^28^ |
| Sanquin homeservice | €200 per administration | Gamma (40.00) || | Estimated by Sanquin, personal communication MdH |
| Advanced fetal ultrasound | €851.48 | Gamma (42.57) † | ^29^ assuming the highest rate; costs updated to 2022 using Dutch CPI |
| Standard fetal ultrasound | €166.66 | Gamma (8.33) † | ^29^ costs updated to 2022 using Dutch CPI |
| Consult gynaecologist | €185.87 | Gamma (9.29) † | Manual for cost research. ^28^ |
| Consult midwife | €31.54 | Gamma (3.17) ‡ | Manual for cost research. ^28^ |
| *Postnatal treatment* | | | |
| HPA matched platelet transfusion | €365.37 | Gamma (17.65) † | Sanquin, personal communication TWdV |
| Cranial ultrasound | €100.35 | Gamma (5.02) † | 30 |
| Admission maternal ward (day) | €449.86 | Gamma (44.99) ‡ | 30 |
| Admission high care neonatology | €1830.87 | gamma (183.09) ‡ | 30 |
| *Lifetime costs per health state* | | | |
| Healthy state | €0 | NA | - |
| Not disabled state | €0 | NA | - |
| Disabled state (excl. informal costs) | €802,868 |  | 31 |
| Lifetime informal care costs (disabled state) | €340,999 |  | 32 |
| Total lifetime costs disabled health state | €1,143,867 | Gamma (571,933.62)# | 30, 31 |
| Death state | €0 |  | - |
| † SE of 5%. ‡ SE of 10%. § SE of 15%. || SE of 20%. # SE of 50%.  SE, standard error; NaCl, natrium chloride; ml, mililitre; CPI, consumer price index; HPA, human platelet antigen; NA, not applicable; excl., excluding. | | | |

## Supplemental Table 5: Utility, life expectancy and QALY

| **Parameter name** | **Value** | **Distribution**  **Gamma (SE)** | **Source** |
| --- | --- | --- | --- |
| *Utility per health state* | | | |
| Dead | 0 | N/A | By definition |
| Disabled | 0.550 | Beta (0.110)|| | 33-36 |
| Not disabled | 0.910 | Beta (0.046) † | 37 |
| Healthy | 0.910 | Beta (0.046) † | 37 |
| *Life expectancy per health state* | | | |
| Dead | 0 | N/A | By definition |
| Disabled | 50 | Gamma (10)|| | 38 |
| Not disabled | 81.66 | Gamma (4.083) † | 39 |
| Healthy | 81.66 | Gamma (4.083) † | 39 |
| † SE of 5%. ‡ SE of 10%. § SE of 15%. || SE of 20%. # SE of 50%. | | | |

| **Health state** | **Value - undiscounted** | **Value - discounted** |
| --- | --- | --- |
| Dead | 0 | 0 |
| Disabled | 27.5 | 19.54 |
| Not disabled | 74.31 | 43.41 |
| Healthy | 74.31 | 43.41 |

## Supplemental Table 6: QALY per health state

# Supplemental Figures

## Supplemental Figure 1: No screening - decision tree

## Supplemental Figure 2: HPA-1a screening - decision tree

## Supplemental Figure 3: One way sensitivity analysis.


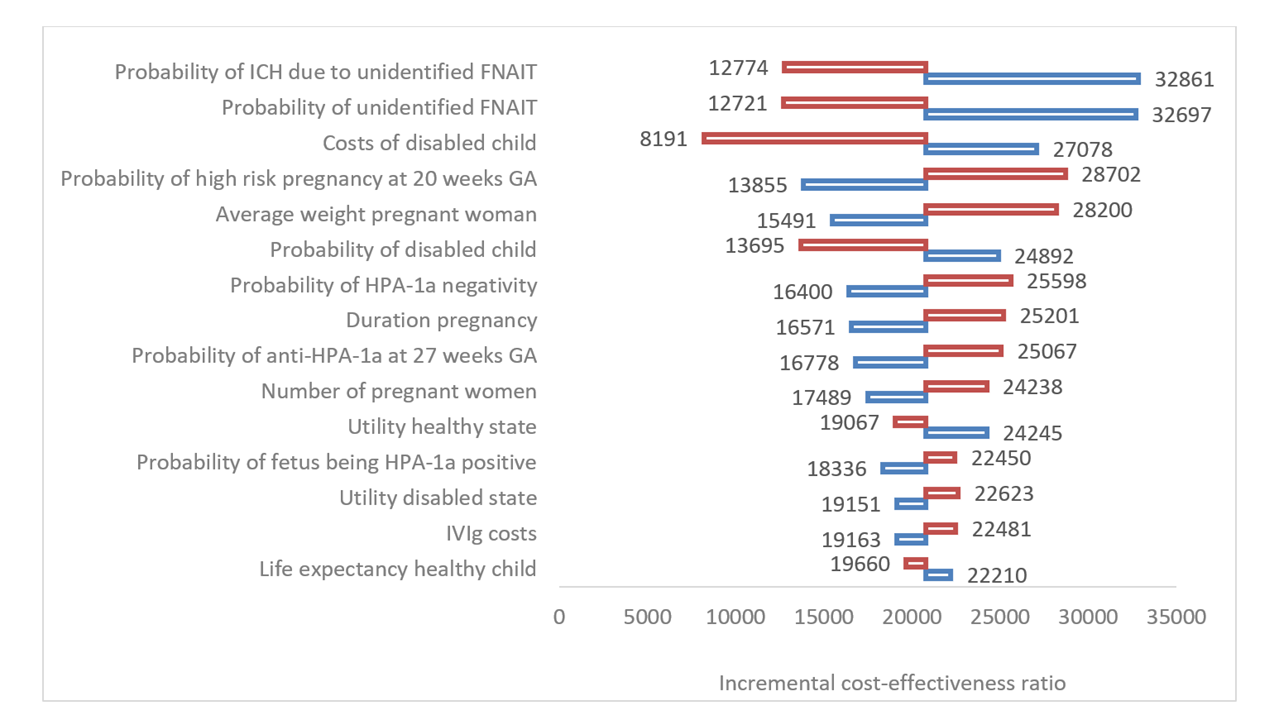


Univariate sensitivity analysis: cost-effectiveness ratio (cost per QALY) for minimum (red bars) and maximum values (blue bars) of the input parameters. Base case ICER €20,782 per QALY (price level 2022).

ICH, intracranial haemorrhage; FNAIT, fetal and neonatal alloimmune thrombocytopenia; GA, gestational age; IVIg, intravenous immune globulins.

# References supplemental data

1. Davoren A, Curtis BR, Aster RH, McFarland JG. Human platelet antigen-specific alloantibodies implicated in 1162 cases of neonatal alloimmune thrombocytopenia. Transfusion. 2004;44(8):1220-5.

2. Husebekk A, Killie MK, Kjeldsen-Kragh J, Skogen B. Is it time to implement HPA-1 screening in pregnancy? Current opinion in hematology. 2009;16(6):497-502.

3. Kjeldsen-Kragh J, Husebekk A, Killie MK, Skogen B. Is it time to include screening for neonatal alloimmune thrombocytopenia in the general antenatal health care programme? Transfusion and apheresis science : official journal of the World Apheresis Association : official journal of the European Society for Haemapheresis. 2008;38(3):183-8.

4. Winkelhorst D, de Vos TW, Kamphuis MM, Porcelijn L, Lopriore E, Oepkes D, et al. HIP (HPA-screening in pregnancy) study: protocol of a nationwide, prospective and observational study to assess incidence and natural history of fetal/neonatal alloimmune thrombocytopenia and identifying pregnancies at risk. BMJ open. 2020;10(7):e034071.

5. Gafni A, Blanchette VS. Screening for neonatal alloimmune thrombocytopenia: an economic perspective. Current studies in hematology and blood transfusion. 1988(54):140-7.

6. Durand-Zaleski I, Schlegel N, Blum-Boisgard C, Uzan S, Dreyfus M, Kaplan C. Screening primiparous women and newborns for fetal/neonatal alloimmune thrombocytopenia: a prospective comparison of effectiveness and costs. Immune Thrombocytopenia Working Group. American journal of perinatology. 1996;13(7):423-31.

7. Turner ML, Bessos H, Fagge T, Harkness M, Rentoul F, Seymour J, et al. Prospective epidemiologic study of the outcome and cost-effectiveness of antenatal screening to detect neonatal alloimmune thrombocytopenia due to anti-HPA-1a. Transfusion. 2005;45(12):1945-56.

8. Killie MK, Kjeldsen-Kragh J, Husebekk A, Skogen B, Olsen JA, Kristiansen IS. Cost-effectiveness of antenatal screening for neonatal alloimmune thrombocytopenia. BJOG : an international journal of obstetrics and gynaecology. 2007;114(5):588-95.

9. Tiller H, Kamphuis MM, Flodmark O, Papadogiannakis N, David AL, Sainio S, et al. Fetal intracranial haemorrhages caused by fetal and neonatal alloimmune thrombocytopenia: an observational cohort study of 43 cases from an international multicentre registry. BMJ open. 2013;3(3).

10. Williamson LM, Hackett G, Rennie J, Palmer CR, Maciver C, Hadfield R, et al. The natural history of fetomaternal alloimmunization to the platelet-specific antigen HPA-1a (PlA1, Zwa) as determined by antenatal screening. Blood. 1998;92(7):2280-7.

11. Kjeldsen-Kragh J, Killie MK, Tomter G, Golebiowska E, Randen I, Hauge R, et al. A screening and intervention program aimed to reduce mortality and serious morbidity associated with severe neonatal alloimmune thrombocytopenia. Blood. 2007;110(3):833-9.

12. Versteegh M, Knies S, Brouwer W. From Good to Better: New Dutch Guidelines for Economic Evaluations in Healthcare. PharmacoEconomics. 2016;34(11):1071-4.

13. van der Ploeg CPB, Oomen P, van Lent M. Prenatale Screening Infectieziekten en Erytrocytenimmunisatie (PSIE). 2021.

14. Winkelhorst D, Kamphuis MM, Steggerda SJ, Rijken M, Oepkes D, Lopriore E, et al. Perinatal Outcome and Long-Term Neurodevelopment after Intracranial Haemorrhage due to Fetal and Neonatal Alloimmune Thrombocytopenia. Fetal diagnosis and therapy. 2019;45(3):184-91.

15. Tiller H, Killie MK, Skogen B, Øian P, Husebekk A. Neonatal alloimmune thrombocytopenia in Norway: poor detection rate with nonscreening versus a general screening programme. BJOG : an international journal of obstetrics and gynaecology. 2009;116(4):594-8.

16. de Vos TW, Winkelhorst D, Porcelijn L, Beaufort M, Oldert G, van der Bom JG, et al. The natural history of human platelet antigen (HPA)-1a alloimmunised pregnancies: a prospective observational cohort study. manuscript is currently under review ed2022.

17. Kamphuis MM, Paridaans N, Porcelijn L, De Haas M, Van Der Schoot CE, Brand A, et al. Screening in pregnancy for fetal or neonatal alloimmune thrombocytopenia: systematic review. BJOG : an international journal of obstetrics and gynaecology. 2010;117(11):1335-43.

18. de Vos TW, Porcelijn L, Hofstede-van Egmond S, Pajkrt E, Oepkes D, Lopriore E, et al. Clinical characteristics of human platelet antigen (HPA)-1a and HPA-5b alloimmunised pregnancies and the association between platelet HPA-5b antibodies and symptomatic fetal neonatal alloimmune thrombocytopenia. British journal of haematology. 2021;195(4):595-603.

19. de Vos TW, Winkelhorst D, Árnadóttir V, van der Bom JG, Canals Surís C, Caram-Deelder C, et al. Postnatal treatment for children with fetal and neonatal alloimmune thrombocytopenia: a multicentre, retrospective, cohort study. The Lancet Haematology. 2022;9(11):e844-e53.

20. de Vos TW, de Haas M, Oepkes D, Tan R, van der Schoot CE, Steggerda SJ, et al. Long-term neurodevelopmental outcome in children after antenatal intravenous immune globulin treatment in fetal and neonatal alloimmune thrombocytopenia. American journal of obstetrics and gynecology. 2022.

21. Kjeldsen-Kragh J, Fergusson DA, Kjaer M, Lieberman L, Greinacher A, Murphy MF, et al. Fetal/neonatal alloimmune thrombocytopenia: a systematic review of impact of HLA-DRB3*01:01 on fetal/neonatal outcome. Blood advances. 2020;4(14):3368-77.

22. Killie MK, Husebekk A, Kjeldsen-Kragh J, Skogen B. A prospective study of maternal anti-HPA 1a antibody level as a potential predictor of alloimmune thrombocytopenia in the newborn. Haematologica. 2008;93(6):870-7.

23. Gleadall NS, Veldhuisen B, Gollub J, Butterworth AS, Ord J, Penkett CJ, et al. Development and validation of a universal blood donor genotyping platform: a multinational prospective study. Blood advances. 2020;4(15):3495-506.

24. Timmer TC, de Groot R, Habets K, Merz EM, Prinsze FJ, Atsma F, et al. Donor InSight: characteristics and representativeness of a Dutch cohort study on blood and plasma donors. Vox sanguinis. 2019;114(2):117-28.

25. Diagnostics S. Foetale HPA-1a genotypering in maternaal plasma - Diagnostische testen 2022 [Available from: https://www.sanquin.org/nl/producten-en-diensten/diagnostiek/diagnostische-testen/index/name/t012-foetale-hpa-1a-genotypering-in-maternaal-plasma.

26. Diagnostics S. Trombocytopenie van de pasgeborene (of foetus) 2022 [cited 2022 19-06-2022]. Available from: https://www.sanquin.org/nl/producten-en-diensten/diagnostiek/diagnostische-testen/index/name/t911-trombocytopenie-van-de-pasgeborene-of-foetus.

27. Nederland Z. Medicijnkosten.nl [translated into English: medicinecosts.nl] 2022 [Available from: https://www.medicijnkosten.nl/.

28. Kanters TA, Bouwmans CAM, van der Linden N, Tan SS, Hakkaart-van Roijen L. Update of the Dutch manual for costing studies in health care. PloS one. 2017;12(11):e0187477.

29. LUMC. Passanten prijslijst DBC-zorgproducten en overige zorgproducten jaar 2021.

30. Liem SM, van Baaren GJ, Delemarre FM, Evers IM, Kleiverda G, van Loon AJ, et al. Economic analysis of use of pessary to prevent preterm birth in women with multiple pregnancy (ProTWIN trial). Ultrasound in obstetrics & gynecology : the official journal of the International Society of Ultrasound in Obstetrics and Gynecology. 2014;44(3):338-45.

31. Kruse M, Michelsen SI, Flachs EM, Brønnum-Hansen H, Madsen M, Uldall P. Lifetime costs of cerebral palsy. Developmental medicine and child neurology. 2009;51(8):622-8.

32. Mitchell LA, Hirdes J, Poss JW, Slegers-Boyd C, Caldarelli H, Martin L. Informal caregivers of clients with neurological conditions: profiles, patterns and risk factors for distress from a home care prevalence study. BMC health services research. 2015;15:350.

33. Jarl J, Alriksson-Schmidt A, Rodby-Bousquet E. Health-related quality of life in adults with cerebral palsy living in Sweden and relation to demographic and disability-specific factors. Disability and health journal. 2019;12(3):460-6.

34. Macedo AF, Ramos PL, Hernandez-Moreno L, Cima J, Baptista AMG, Marques AP, et al. Visual and health outcomes, measured with the activity inventory and the EQ-5D, in visual impairment. Acta ophthalmologica. 2017;95(8):e783-e91.

35. Langelaan M, de Boer MR, van Nispen RM, Wouters B, Moll AC, van Rens GH. Impact of visual impairment on quality of life: a comparison with quality of life in the general population and with other chronic conditions. Ophthalmic epidemiology. 2007;14(3):119-26.

36. Kirkham FJ, Vigevano F, Raspall-Chaure M, Wilken B, Lee D, Le Reun C, et al. Health-related quality of life and the burden of prolonged seizures in noninstitutionalized children with epilepsy. Epilepsy & behavior : E&B. 2020;102:106340.

37. Janssen MF, Szende A, Cabases J, Ramos-Goñi JM, Vilagut G, König HH. Population norms for the EQ-5D-3L: a cross-country analysis of population surveys for 20 countries. The European journal of health economics : HEPAC : health economics in prevention and care. 2019;20(2):205-16.

38. Strauss D, Brooks J, Rosenbloom L, Shavelle R. Life expectancy in cerebral palsy: an update. Developmental medicine and child neurology. 2008;50(7):487-93.

39. Statline C. Levensverwachting leeftijd in jaren 2022 [cited 2022 19-06-2022]. Available from: https://opendata.cbs.nl/statline/#/CBS/nl/dataset/37360ned/table?fromstatweb.

40. Maslanka K, Guz K, Zupanska B. Antenatal screening of unselected pregnant women for HPA-1a antigen, antibody and alloimmune thrombocytopenia. Vox sanguinis. 2003;85(4):326-7.

41. Blanchette VS, Chen L, de Friedberg ZS, Hogan VA, Trudel E, Décary F. Alloimmunization to the PlA1 platelet antigen: results of a prospective study. British journal of haematology. 1990;74(2):209-15.

42. Bessos H, Killie MK, Seghatchian J, Skogen B, Urbaniak SJ. The relationship of anti-HPA-1a amount to severity of neonatal alloimmune thrombocytopenia - Where does it stand? Transfusion and apheresis science : official journal of the World Apheresis Association : official journal of the European Society for Haemapheresis. 2009;40(2):75-8.

43. Fretheim A. Cost-effectiveness analysis of screening for neonatal alloimmune thrombocytopenia was based on invalid assumption. BJOG : an international journal of obstetrics and gynaecology. 2008;115(3):412-3; author reply 3-4; discussion 4.

44. Cameron D, Ubels J, Norström F. On what basis are medical cost-effectiveness thresholds set? Clashing opinions and an absence of data: a systematic review. Global health action. 2018;11(1):1447828.

45. Bussel JB, Vander Haar EL, Berkowitz RL. New developments in fetal and neonatal alloimmune thrombocytopenia. American journal of obstetrics and gynecology. 2021;225(2):120-7.

46. Kapur R, Kustiawan I, Vestrheim A, Koeleman CA, Visser R, Einarsdottir HK, et al. A prominent lack of IgG1-Fc fucosylation of platelet alloantibodies in pregnancy. Blood. 2014;123(4):471-80.

47. Santoso S, Wihadmadyatami H, Bakchoul T, Werth S, Al-Fakhri N, Bein G, et al. Antiendothelial αvβ3 Antibodies Are a Major Cause of Intracranial Bleeding in Fetal/Neonatal Alloimmune Thrombocytopenia. Arteriosclerosis, thrombosis, and vascular biology. 2016;36(8):1517-24.

48. Chitty LS, Finning K, Wade A, Soothill P, Martin B, Oxenford K, et al. Diagnostic accuracy of routine antenatal determination of fetal RHD status across gestation: population based cohort study. BMJ (Clinical research ed). 2014;349:g5243.

49. Winkelhorst D, Porcelijn L, Muizelaar E, Oldert G, Huiskes E, van der Schoot CE. Fast and low-cost direct ELISA for high-throughput serological HPA-1a typing. Transfusion. 2019;59(9):2989-96.

50. Tariefbeschikking. Tariefbeschikking 2022 [Available from: http://www.pns.nl/documenten/tariefbeschikking-2022.
